# Perceived Maternal Overprotection and Amygdala Functional Connectivity in Children: Implications for Childhood Depressive Symptoms

**DOI:** 10.1155/da/4516539

**Published:** 2026-08-26

**Authors:** Lizhu Luo, Ai Peng Tan, Yap Seng Chong, Peter Gluckman, Johan Gunnar Eriksson, Shi Yu Chan, Pei Huang, Juan Helen Zhou, Marielle V. Fortier, Michael J. Meaney

**Affiliations:** ^1^ School of Social Sciences, Nanyang Technological University, Singapore, Singapore, ntu.edu.sg; ^2^ Institute for Human Development and Potential, A*STAR Research Entities, Singapore, Singapore; ^3^ Yong Loo Lin School of Medicine, National University of Singapore, Singapore, Singapore, nus.edu.sg; ^4^ Department of Diagnostic Imaging, National University Health System, Singapore, Singapore, nuhs.edu.sg; ^5^ Liggins Institute, University of Auckland, Auckland, New Zealand, auckland.ac.nz; ^6^ Department of General Practice and Primary Health Care, University of Helsinki, Helsinki, Finland, helsinki.fi; ^7^ Department of Electrical and Computer Engineering, National University of Singapore, Singapore, Singapore, nus.edu.sg; ^8^ Department of Diagnostic and Interventional Imaging, KK Women’s and Children’s Hospital, Singapore, Singapore, kkh.com.sg; ^9^ Department of Psychiatry, Douglas Mental Health University Institute, McGill University, Montreal, Canada, mcgill.ca

**Keywords:** amygdala, children, depressive symptoms, maternal overprotection, resting-state functional connectivity (rsFC)

## Abstract

**Background:**

Parenting significantly influences children’s emotional well‐being, but the specific nature and neural mechanisms of this influence remain unclear. This study investigated (1) the association between perceived maternal parenting and amygdala resting‐state functional connectivity (rsFC) in childhood and (2) whether amygdala rsFC moderates the relation between perceived maternal parenting and subsequent depressive symptoms.

**Methods:**

A total of 220 children from the Growing Up in Singapore Towards Healthy Outcomes (GUSTO) cohort underwent resting‐state fMRI at age 7.5 and assessments of perceived maternal parenting and depressive symptoms at ages 8.5–10.5 using the Children’s Depression Inventory (CDI‐2) and the child‐reported Parental Bonding Instrument (PBI). Multiple regression analyses explored associations between maternal parenting (PBI subscales: overprotection and care) and rsFC of the whole amygdala and subnuclei. Moderation analyses evaluated whether amygdala rsFC moderated the relation between maternal parenting and subsequent depressive symptoms in children.

**Results:**

Two neural pathways were identified in relation to perceived maternal overprotection. One pathway showed a positive association between the rsFC of the right (R) basolateral amygdala (BLA) and the R angular gyrus (AG) as well as the left (L) inferior parietal gyrus (IPG). The other pathway showed a negative association with the L superficial amygdala (SFA)–anterior insula (INS) rsFC. The pathway from R amygdala–AG rsFC significantly moderated the strong positive association between perceived maternal overprotection and subsequent depressive symptoms. These associations were independent of maternal depressive symptoms.

**Conclusions:**

This study provides insight into the neurobiological basis linking perceived maternal parenting to subsequent depressive symptoms. R amygdala–AG rsFC may serve as a neural marker of variability in children’s responses to perceived maternal overprotection, shaping the nature of their depressive symptoms.

## 1. Introduction

Major depressive disorder (MDD) is one of the most prevalent mental health issues affecting children and adolescents worldwide [1] as well as in Singapore [2]. Parenting is an important factor affecting child development, including neural circuits underlying emotional development and mental health outcomes [3]. An association between parenting, especially maternal parenting, and depressive symptoms in offspring has been widely reported [4]. This relation is predicted from attachment theory [5] such that the mother–child relationship stands as one of the most profound and intimate social bonds in humans.

Within the multifaceted landscape of parenting, Parker et al. [6] developed the Parental Bonding Instrument (PBI), a self‐report measure designed to assess perceived parenting in offspring. The PBI has proven to be a highly validated and reliable tool, widely used not only in adults [7] but also in children and adolescents aged 7–18 years [8]. The PBI delineates two distinct dimensions: care and overprotection/control. Research using the PBI shows that parenting styles with low care or high overprotection are associated with more mental health issues, especially depressive symptoms, when it comes to perceived maternal parenting [9, 10]. This pattern is evident across various cultures, where perceived maternal overprotection has been consistently linked to an increased risk of depression [4].

Maternal parenting influences brain development, with harsh maternal parenting linked to smaller amygdala volumes in adolescents [11]. The amygdala, a key brain region for emotional processing, has emerged as critically implicated in both the effects of maternal parenting and the development of depression [12, 13]. High perceived maternal warmth and support is linked to reduced amygdala activation to fearful expressions in adolescents [13], while greater perceived maternal overprotection in childhood is associated with increased amygdala responses to threatening stimuli in adulthood [14]. However, due to the complex interplay between emotion and cognition through brain connectivity [15], research on the impact of perceived maternal parenting on children’s amygdala function, particularly amygdala‐based functional networks, remains limited.

Studies in adolescents have linked maternal aggression and hostility to functional connections between the amygdala and regions such as the insula (INS), temporal gyrus, and visual cortex [16], as well as the frontal and parietal regions [17]. Additionally, less positive parenting has been associated with amygdala functional connectivity to the subgenual anterior cingulate cortex [18]. However, the amygdala consists of several subnuclei, each with unique patterns of connectivity and function. For example, the centromedial amygdala (CMA) is involved in response initiation and allocation of attention [19], the basolateral amygdala (BLA) in sensory processing and fear learning [20], and the superficial amygdala (SFA) in social and affective processing [21]. Despite these established distinctions, there is limited research on how perceived maternal parenting—particularly maternal overprotection and low care—affects the functional connectivity of amygdala subnuclei and influences depressive symptoms in children.

**Figure 1:**
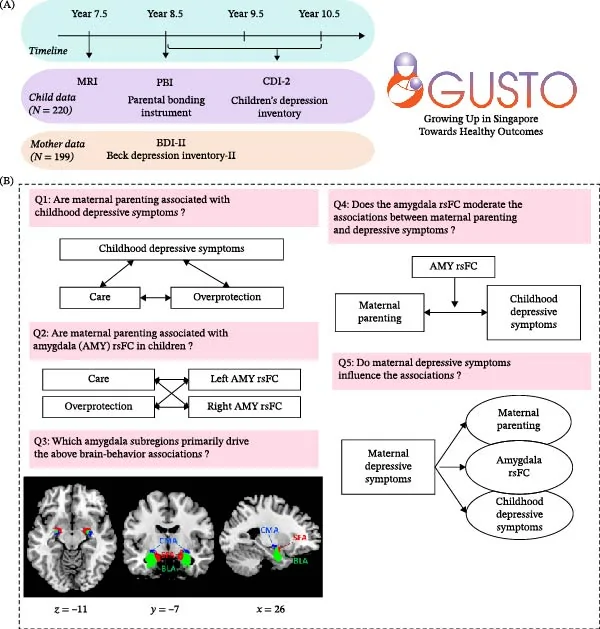
Study outline. (A) Timeline of data collection and measurements. (B) Research questions and ROI masks of amygdala subregions. AMY, amygdala; CMA, centromedial amygdala; BLA, basolateral amygdala; SFA, superficial amygdala; rsFC, resting‐state functional connectivity.

Using data from the Growing Up in Singapore Towards Healthy Outcomes (GUSTO) project, a comprehensive longitudinal birth cohort study based in Singapore, we examined the relation between perceived maternal overprotection and care, as assessed by child‐reported PBI scales, and resting‐state functional connectivity (rsFC) of the whole and subregional amygdala (Figure 1). We also explored how parental factors are associated with subsequent depressive symptoms in children. We hypothesized that perceived high overprotection and low care would correspond with alterations in amygdala rsFC, characterized by increased connectivity with cognitive regions and reduced connectivity with emotional regions, reflecting greater cognitive demands and challenges with negative emotion regulation. Given the central role of the amygdala in maternal parenting and depression, we further hypothesized that amygdala‐based rsFC would moderate the relation between perceived maternal overprotection/low care in children and their subsequent depressive symptoms. Finally, recognizing that maternal depression can influence parenting and is linked to amygdala connectivity in children [22–24], we conducted a secondary analysis to examine any direct effects of maternal mood.

## 2. Method

### 2.1. Participants

Only a total of 220 children from the GUSTO cohort were included in the current study because these participants had complete data across all measures of interest. Specifically, they (1) completed resting‐state fMRI scanning at the age of 7.5 years (*M* = 7.44, SD = 0.13, and age range = 7.25–7.88; 117 girls), (2) completed a self‐reported assessment of maternal parenting at the age of 8.5 years (*M* = 8.89, SD = 0.07, and age range = 8.71–9.22), and (3) provided self‐reported depressive symptom measures at approximately ages 8.5, 9.5, or 10.5 years (*M* = 9.03, SD = 0.37, and age range = 8.71–10.52). Among the 220 children included in the study, maternal depressive symptom data were available for 197 mothers when the children were around 8.5 years old (*M* = 8.88, SD = 0.07, and age range = 8.71–9.22). Participants without complete data on resting‐state fMRI, parenting measures, or depressive symptom assessments were excluded from the present analyses. Figure 1A illustrates the participant flow and data availability.

Ethical approval for the GUSTO study was granted by the National Healthcare Group Domain Specific Review Board (D/2009/021 and B/2014/00411) and the SingHealth Centralized Institutional Review Board (D/2018/2767 and A/2019/2406). This study was conducted in accordance with the Declaration of Helsinki. Written informed consent was obtained from the guardians of all participating children in this study.

### 2.2. Measurements

PBI for Children (PBI‐C) [25]. We employed the 25‐item PBI‐C, a modified version of the PBI [10], to assess perceived maternal parenting in children at the age of 8.5 years. Children were instructed to focus on their mother’s behavior during the past year. Each item presented respondents with four response options: 0 (not true), 1 (somewhat not true), 2 (true), or 3 (very true). The PBI encompasses two distinct dimensions, care (12 items) and overprotection (13 items), which were used to categorize parental bonding styles into four quadrants: optimal parenting (high care/low protection), affectionate constraint (high care/high protection), neglectful parenting (low care/low protection), and affectionless control (low care/high protection). Our focus was on examining how subtle variations in maternal parenting may manifest in a continuous rather than categorical manner within the brain. Consequently, we employed dimensional indices of care and overprotection instead of categorical quadrant assignments.

Children’s Depression Inventory (CDI‐2) [26]: We employed the 28‐item CDI‐2 to evaluate the extent of depressive symptoms among the child participants at ages 8.5, 9.5, or 10.5 years. Each item offered three response choices, representing varying degrees of symptoms: 0 (no symptoms), 1 (mild or probable symptoms), or 2 (definite symptoms). In this study, we focused exclusively on the total CDI scores.

Beck Depression Inventory‐II (BDI‐II) [27]: The 21‐item BDI was employed to assess maternal depressive symptoms when the child participants at ages 8.5 years. Each item offers four answer choices of varying intensity, scored from 0 to 3. In the current study, the total score was computed, with higher scores indicating greater severity of depressive symptoms.

### 2.3. MRI Acquisition

Brain images were obtained at the age of 7.5 years using a 3T scanner (Magnetom Prisma; Siemens, Germany) situated at the Clinical Imaging Research Center of the National University of Singapore. Acquisition of 3D T1‐weighted MPRAGE images adhered to specific imaging parameters: repetition time = 2000 ms, echo time = 2.08 ms, 1 mm isotropic voxels, matrix size = 192 × 192, and number of slices = 192. Additionally, one run of single‐shot echo‐planar resting‐state fMRI was collected employing the following parameters: 5.24 min (120 time points), repetition time = 2620 ms, echo time = 27 ms, flip angle = 90°, 3 mm isotropic voxels, matrix = 64 × 64, and 48 interleaved axial slices with no gap.

### 2.4. MRI Data Preprocessing

We conducted resting‐state fMRI data preprocessing using the default pipeline in the CONN toolbox (version 20b) [28] implemented in Statistical Parametric Mapping (SPM12; www.fil.ion.ucl.ac.uk/spm) in Matlab (R2020a, MathWorks, Inc., USA). Preprocessing included functional realignment and unwarping, slice‐timing correction, outlier identification, direct segmentation and normalization, and spatial smoothing. Specifically, motion correction was performed during realignment, which estimates six rigid‐body motion parameters (three translations and three rotations). Outlier volumes were identified using the artifact detection tools (ART) implemented in CONN, with thresholds of framewise displacement > 0.9 mm and global BOLD signal changes > 5 standard deviations. Next, functional and anatomical data were normalized into standard Montreal Neurological Institute (MNI) space and segmented into gray matter, white matter, and CSF tissue classes. Subsequently, the functional data underwent smoothing through spatial convolution with a 6 mm FWHM Gaussian kernel. Denoising was then performed using an ordinary least squares regression model that included the six motion parameters, regressors for outlier volumes (scrubbing), white matter and cerebrospinal fluid signals, and linear trends [29]. A temporal band‐pass filter (0.008–0.09 Hz) was applied following regression. Only participants with motion below specific thresholds (maximum motion < 6 mm, mean motion < 0.6 mm, and fewer than 20% outlier scans) were included in the analysis (220 children).

Following preprocessing, we conducted amygdala functional connectivity analysis. For each subject, separate seed‐based connectivity (SBC) maps were generated based on individual amygdala regions of interest (ROIs), calculating Fisher‐transformed bivariate correlation coefficients of the BOLD time series using a hemodynamic response factor‐weighted general linear model.

### 2.5. ROI Definition

The study encompassed a total of eight ROIs in the amygdala, comprising the complete left (L) and right (R) amygdala structures and their corresponding subregions: CMA, BLA, and SFA. In line with our previous study [30], these subregions were initially defined using cytoarchitectonic probability maps [31] at a probability threshold of 60%, as implemented in the SPM Anatomy Toolbox 1.8 [32]. Subsequently, nonoverlapping amygdala subregions were generated following the methodology outlined in a prior publication [33]. Specifically, voxels exceeding this threshold were considered for inclusion in the corresponding subregions (CMA, BLA, and SFA). In cases where a voxel exceeded the threshold in more than one subdivision, it was uniquely assigned to the subregion for which it showed the highest probability value, resulting in mutually exclusive masks. Voxels that did not exceed the threshold in any subdivision were excluded from further analysis. The spatial characteristics of the amygdala subregions are presented in Table S1, and their spatial distributions are shown in Figure 1B, where all subregions are overlaid on a template using MRIcron (https://www.nitrc.org/projects/mricron/) and displayed in different colors.

### 2.6. Statistical Analysis

Statistical analyses of the behavioral measures and demographic variables were performed using SPSS Statistics 26 (Armonk, NY: IBM Corp.). To verify the association between maternal parenting and subsequent depressive symptoms, we employed Spearman correlation analyses to assess the relation between two PBI subscale scores and CDI total scores.

To investigate the association between perceived maternal parenting and the whole amygdala rsFC in children, we conducted separate multiple regression analyses using SPM12 on the rsFC of the R and L entire amygdala regions. In these analyses, maternal overprotection and lack of maternal care (negative values on the care dimension) were examined as variables while controlling for each other and covariates including sex, ethnicity, age at MRI scanning, and age at PBI assessment. We employed t‐contrasts to examine brain regions showing significantly positive or negative associations between amygdala rsFC and maternal parenting. To control for multiple comparisons, we applied a Bonferroni‐adjusted cluster‐level threshold of *p* < 0.0125 (family‐wise error [FWE]–corrected; *p* < 0.05/4; voxel‐level *p* < 0.001 uncorrected) across four primary whole‐amygdala seed‐based analyses (L/R amygdala × maternal overprotection/care). Significant regions were labeled using the automated anatomical labeling atlas 3 (AAL3) [34].

Subsequent subregional analyses of the CMA, BLA, and SFA were conducted as follow‐up analyses at the whole‐brain voxel‐wise level. These analyses were intended to characterize which amygdala subregions contributed to the effects already detected in the whole‐amygdala analyses. To control for multiple comparisons across the three subregions within each hemisphere, an additional Bonferroni‐corrected cluster‐level threshold of *p* < 0.017 (FWE‐corrected; *p* < 0.05/3; voxel‐level *p* < 0.001 uncorrected) was applied separately to the L and R amygdala analyses. To assess robustness across parcellation schemes, we conducted a sensitivity analysis using an alternative connectivity‐based Brainnetome Atlas (BNA) [35], which defines medial and lateral amygdala divisions. Spatial comparison indicated partial correspondence between atlases, with the BLA overlapping with the lateral division and the SFA overlapping with the medial division (see Figure S1).

To visualize the significant associations more clearly, we generated scatter plots illustrating the relationship between connectivity measures and PBI scores using the ggplot package in R (version 4.2.2). The method for quantifying connectivity measurements involved using the MarsBaR toolbox [36] within SPM. This approach entailed centering a 6‐mm‐radius sphere on the peak coordinates of statistically significant brain regions and subsequently extracting parameter estimates from within the sphere.

To examine whether observed amygdala rsFC might moderate the relation between maternal parenting and subsequent depressive symptoms assessed 1–3 years after MRI acquisition, we conducted moderation analyses using the PROCESS macro for SPSS v4.2 [37]. To ascertain whether the associations identified in this study could be influenced by maternal mood, we conducted Spearman correlation analyses to examine the relation between maternal BDI scores and the PBI scores, CDI scores, and rsFC values of the children.

## 3. Results

### 3.1. Perceived Maternal Parenting and Childhood Depressive Symptoms

We first calculated the average total scores for the two PBI subscales (maternal overprotection, *M* = 17.7, SD = 5.3; maternal care, *M* = 26.9, SD = 5.4) and the CDI total symptom score (*M* = 10.8, SD = 6.9). We also conducted an analysis to determine sex differences in these variables, which yielded no significant findings (*p* > 0.1 for all cases; see Table 1).

**Table 1:** Descriptive statistics and sex differences.

Variable	Girls			Boys			Differences	
	*N*	Mean	SD	*N*	Mean	SD	*t*	*p*
Child age at MRI scan (years)	117	7.438	0.132	103	7.44	0.132	−0.079	0.937
Ethnicity								
Chinese	69	—	—	52	—	—	—	—
Malaysian	33	—	—	35	—	—	—	—
Indian	15	—	—	16	—	—	—	—
Children’s Depression Inventory, CDI‐2								
Child age at completion (years)	117	9.053	0.399	103	9.001	0.327	1.044	0.297
Total score	—	11.299	6.846	—	10.194	6.982	1.184	0.238
Parental Bonding Instrument for Children, PBI‐C								
Child age at completion (years)	117	8.888	0.081	103	8.884	0.065	0.405	0.686
Maternal overprotection	—	17.222	5.392	—	18.165	5.208	−1.315	0.19
Maternal care	—	26.735	5.299	—	27.097	5.43	−0.5	0.618
Beck Depression Inventory‐II, BDI‐II (for mothers)								
Child age at completion (years)	107	8.885	0.082	92	8.88	0.063	0.438	0.662
Total score^a^	—	6.29	7.471	—	6.891	7.161	−0.577	0.564

^a^This is the only score reflecting maternal status, which pertains to self‐reported maternal depressive symptoms. All other data in the table are related to the child’s status.

**Figure 2:**
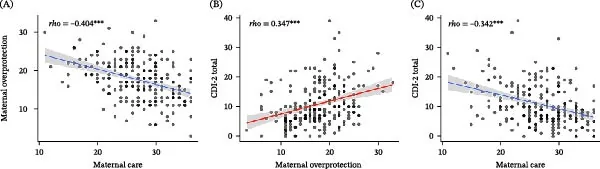
Associations between perceived maternal overprotection, maternal care, and childhood depressive symptoms. (A) Negative associations between perceived maternal overprotection and maternal care. (B) Positive associations between perceived maternal overprotection and CDI total scores. (C) Negative associations between perceived maternal care and CDI total scores. CDI‐2, Children’s Depression Inventory; *r*, coefficients; confidence interval (CI), 95%;  ^∗∗∗^ *p* < 0.001.

We then evaluated the associations between perceived maternal overprotection, maternal care, and subsequent depressive symptoms. Given the non‐normal distribution of CDI scores (Shapiro–Wilk test, *p* < 0.001), Spearman correlation analyses were conducted. We found a significant negative association between maternal care and maternal overprotection (rho = −0.404, *p* < 0.001; Figure 2A). Additionally, maternal overprotection showed a positive correlation (rho = 0.347, *p* < 0.001; Figure 2B), whereas maternal care exhibited a negative correlation (rho = −0.342, *p* < 0.001; Figure 2C) with childhood depressive symptoms.

### 3.2. Perceived Maternal Parenting and Amygdala rsFC

**Figure 3:**
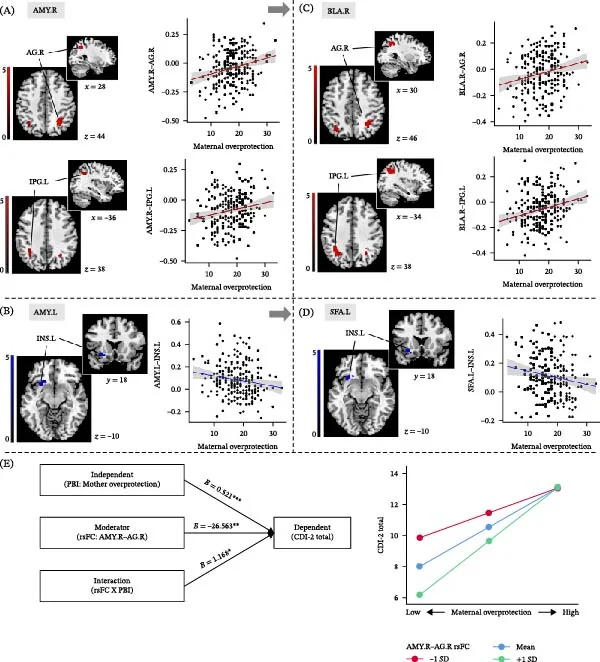
Associations between amygdala functional connectivity and perceived maternal overprotection and the moderating effect on childhood depressive symptoms. (A) Positive associations with the functional connectivity between right amygdala and right angular gyrus as well as left inferior parietal gyrus. (B) Negative associations with the left amygdala and left anterior insula connectivity. (C) Positive associations with the functional connectivity between right basolateral amygdala and right angular gyrus as well as left inferior parietal gyrus. (D) Negative associations with the left superficial amygdala and left anterior insula connectivity. (E) The moderation model of the functional connectivity between the right whole amygdala and right angular gyrus on the association and the visualization of the moderating effect splitting maternal overprotection scores and rsFCs into low (mean −1 SD), medium (mean), and high (mean +1 SD) categories. AMY, amygdala; SFA, superficial amygdala; BLA, basolateral amygdala; AG, angular gyrus; IPG, inferior parietal gyrus; INS, insula; L, left; R, right; *x*/*y*/*z*, peak MNI coordinate; B, coefficients; CDI‐2, Children’s Depression Inventory; rsFC, resting‐state functional connectivity; confidence interval (CI), 95%;  ^∗^ *p* < 0.05;  ^∗∗^ *p* < 0.01;  ^∗∗∗^ *p* < 0.001.

We examined associations of variations in maternal parenting and amygdala rsFC using separate multiple regression analyses for the entire L and R amygdala with correction for multiple comparisons (see Section 2). Our investigation revealed significant results at the whole‐brain level (Table 2). First, we observed significant positive associations between maternal overprotection and the rsFC of the R amygdala with the R angular gyrus (AG) (*k* = 197, *t* = 5.03, *p*_FWE_ = 0.001, and *x*/*y*/*z*: 28/−58/44; Figure 3A) as well as L inferior parietal gyrus (IPG) (*k* = 143, *t* = 4.32, *p*_FWE_ = 0.008, and *x*/*y*/*z*: −36/−48/38; Figure 3A). Second, our analyses revealed a negative correlation between maternal overprotection and the rsFC of the L amygdala and L anterior INS (*k* = 140, *t* = 4.85, *p*_FWE_ = 0.008, and *x*/*y*/*z*: −28/18/−10; Figure 3B). In sum, the results indicate significant associations between maternal overprotection and increased rsFC between the R amygdala and the R AG/L IPG, along with decreased rsFC between the L amygdala and the L anterior INS. We did not identify any significant association between maternal care and bilateral amygdala rsFC at the whole‐brain level.

**Table 2:** Significant associations between maternal overprotection and amygdala‐seeded functional connectivity.

Seed	Region	*k*	*t*	*p*_FWE_	MNI coordinates		
					*x*	*y*	*z*
Positive associations							
R amygdala	R angular gyrus	197	5.03	0.001	28	−58	44
	L inferior parietal gyrus	143	4.32	0.008	−36	−48	38
R basolateral amygdala	R angular gyrus	192	4.58	0.001	30	−52	46
	L inferior parietal gyrus	303	4.59	<0.001	−34	−52	38
Negative associations							
L amygdala	L anterior insula	140	4.85	0.008	−28	18	−10
L superficial amygdala	L anterior insula	127	4.63	0.012	−28	18	−10

*Note: k*, cluster size; *p*, family‐wise error (FWE) corrected at the cluster level.

Abbreviation: MNI, Montreal Neurological Institute.

### 3.3. Amygdala Subregions Driving the Associations

We then sought to identify the specific subregions of the amygdala accounting for the associations with maternal overprotection. Separate multiple regression analyses were conducted for the rsFC of L/R CMA, BLA, and SFA subregions with correction for multiple comparisons (see Section 2). Significant results emerged for the L SFA and R BLA rsFC at the whole‐brain level (Table 2). Specifically, the increased rsFC in the R amygdala as a function of maternal overprotection was predominantly driven by the rsFC between the R BLA and the R AG (*k* = 192, *t* = 4.58, *p*_FWE_ = 0.001, and *x*/*y*/*z*: 30/−52/46; Figure 3C) as well as L IPG (*k* = 303, *t* = 4.59, *p*_FWE_ < 0.001, and *x*/*y*/*z*: −34/−52/38; Figure 3C). No significant associations with maternal overprotection and amygdala subregions were observed for the increased rsFC between the R SFA/CMA and R AG/L IPG. In contrast, the association between increased maternal overprotection and the reduced rsFC in the L amygdala was primarily driven by the rsFC between the L SFA and the L anterior INS (*k* = 127, *t* = 4.63, *p*_FWE_ = 0.012, and *x*/*y*/*z*: −28/18/−10; Figure 3D). No significant associations with maternal overprotection were observed for the L BLA/CMA subregions and the L INS.

Sensitivity analyses using connectivity‐based amygdala subregions from the BNA yielded largely consistent results. The positive association between maternal overprotection and R lateral amygdala rsFC with the R AG (*k* = 313, *t* = 4.82, *p*_FWE_ < 0.001, and *x*/*y*/*z*: 28/−58/44) and L IPG (*k* = 178, *t* = 3.96, *p*_FWE_ = 0.001, and *x*/*y*/*z*: −32/−40/40) was replicated. The negative association with the L medial amygdala and INS rsFC did not survive whole‐brain correction but remained significant under small‐volume correction using both BNA (*k* = 26, *t* = 4.08, and peak *p*_FWE_ = 0.021) and the AAL atlas (*k* = 30, *t* = 4.08, and peak *p*_FWE_ = 0.023), with identical peak coordinates (*x*/*y*/*z*: −28/18/−10).

### 3.4. Amygdala rsFC Moderates Perceived Maternal Overprotection and Subsequent Depressive Symptoms

We did not identify any significant direct associations between observed amygdala rsFC measures in early childhood and depressive symptoms assessed 1–3 years later (*p* > 0.1). Subsequently, moderation analyses were conducted to investigate the influence of the amygdala rsFC (moderator) on the relation between maternal overprotection (independent variable) and subsequent depressive symptoms (dependent variable) in children. All variables were treated as continuous variables in the model. The results (Figure 3E) revealed a statistically significant total model with the rsFC between the R amygdala and the R AG acting as the moderator (*R* ^2^ = 0.151, *F*_3,216_ = 12.784, and *p* < 0.001). There was a significant interaction effect between maternal overprotection and the R amygdala–AG rsFC (Δ*R* ^2^ = 0.021, *B* = 1.168, and *p* = 0.022), indicating that this rsFC moderated the association between perceived maternal overprotection and subsequent depressive symptoms.

To facilitate visualization of the interaction, conditional effects were plotted at −1 SD, mean, and +1 SD values of rsFC, following the common practice in moderation analysis. Simple slope analyses indicated that maternal overprotection was positively associated with subsequent depressive symptoms at low (−1 SD;*B* = 0.301, SE = 0.114, *t* = 2.64, *p* = 0.009, and 95% CI [0.076, 0.525]), mean (*B* = 0.478, SE = 0.084, *t* = 5.68, *p* < 0.001, and 95% CI [0.312, 0.644]), and high (+1 SD; *B* = 0.655, SE = 0.114, *t* = 5.74, *p* < 0.001, and 95% CI [0.430, 0.880]) levels of rsFC. The strength of this association increased as rsFC increased (Figure 3E). A Johnson–Neyman analysis indicated that the association between maternal overprotection and subsequent depressive symptoms became significant when rsFC values exceeded −0.23, encompassing ~90.45% of the observed sample distribution. No significant moderating effects were observed for the other identified whole or regional amygdala rsFC measures.

We conducted additional sensitivity analysis to evaluate whether the MRI–CDI assessment interval (*M* = 1.59 years, SD = 0.38, and range = 1.02–3.17 years) influenced the moderation findings. Specifically, we reran the moderation model, including the MRI–CDI interval as an additional covariate. The results remained significant, with a significant overall model (*R* ^2^ = 0.160, *F*_4,215_ = 10.267, and *p* < 0.001) and a significant interaction between maternal overprotection and R amygdala–AG rsFC (Δ*R* ^2^ = 0.021, *B* = 1.188, and *p* = 0.020), suggesting that the primary moderation finding was robust to adjustment for the time interval between MRI acquisition and CDI‐2 assessment.

### 3.5. the Influence of Maternal Depressive Symptoms

Given the previous findings showing maternal depression association with both parental care and amygdala connectivity [22–24], we sought to assess whether the associations observed here might be secondary to the influence of maternal mood. Spearman correlation analyses (*N* = 197) revealed no significant correlations between maternal depressive symptoms and measures of maternal overprotection (*p* = 0.501) and childhood depressive symptoms (*p* = 0.751) nor the observed amygdala rsFC measures (*p* > 0.23 for all cases). These findings suggest that the above‐reported brain–behavior associations were not a direct result of maternal mood.

## 4. Discussion

This study adds to the existing literature by demonstrating a strong association between perceived maternal parenting and depressive symptoms in a large sample of Singaporean children. Importantly, we replicated in children the association between PBI reports of maternal parenting and depressive symptoms that is well‐established in adults. We then explored the potential neural mechanisms underlying the influence of perceived maternal parenting on childhood depressive symptoms. The results revealed a noteworthy association between maternal overprotection and two distinct pathways with the amygdala as the core hub. The first pathway operates through increased functional connectivity of the R amygdala, notably the basolateral segment, with both the R AG and L IPG. The second pathway involves a reduction in the rsFC of the L amygdala, particularly through its superficial subregion to the L anterior INS. The first (/positive) but not the second (/negative) pathway significantly moderated the interplay between maternal overprotection and the levels of self‐reported depressive symptoms in children.

The positive pathway associated with perceived maternal overprotection involved the rsFC of the R amygdala with the AG and IPG. The amygdala plays a critical role in emotional processing and regulation [38] and has been identified as a potential biomarker of differential sensitivity to the influence of the parenting environment on social–emotional outcomes in adolescents [39]. The AG, part of the IPG, functions as a cross‐modal integrative hub involved in semantic processing, comprehension, attention, memory, action, conflict resolution, social cognition, etc.), providing meaning and significance to events within a contextual environment based on prior knowledge and expectations [40]. Stronger connectivity within this circuit may therefore reflect enhanced integration of emotional and contextual cues related to maternal behavior. In this context, higher rsFC may index heightened attunement to maternal overprotection, such that children with stronger connectivity are more likely to perceive and encode these caregiving signals. We suggest that this functional connection may support higher‐order cognitive processing during emotion regulation, particularly in evaluating and interpreting parental behaviors.

The negative pathway involved the L amygdala–anterior INS rsFC, indicating that higher maternal overprotection is related to decreased rsFC between these regions. Prior studies show that decreased functional coupling in these regions was negatively correlated with childhood emotional abuse [41] and was observed during negative emotional processing in depressed adolescents [42]. Moreover, an intervention study suggested that heightened amygdala–INS rsFC may serve as a potential therapeutic biomarker for depression, associated with reduced depressive symptoms in adolescents [43]. Given the critical role of the anterior INS in emotional awareness [44], our findings suggest that increased maternal overprotection may disrupt the amygdala–INS rsFC, potentially affecting emotional self‐awareness and leading to atypical emotional responses.

Further subnuclear analyses revealed that the observed positive pathway was primarily driven by the BLA, while the negative pathway was mainly driven by the SFA. The BLA is involved in cognitive affection such as emotional decoding and memory [45], while the SFA is essential for processing sensory information with social‐affective values [21]. Together with their respective connections to the AG and INS, our findings underscore the cognitive and emotional implications of maternal overprotection at the subnuclear level. Importantly, the direction and spatial distribution of these rsFC effects were consistent across both cytoarchitectonic and connectivity‐based parcellation schemes, indicating that the findings are robust and not driven by a specific atlas definition.

Additionally, we detected that the two neural pathways related to perceived maternal overprotection originate from the L and R hemispheric amygdala, respectively. While both amygdalae are engaged in processing negative emotions, the L amygdala also plays a role in processing positive emotions [46]. Experimental studies involving lesions in mice show that although both amygdalae are essential for fear conditioning, only the R amygdala is ultimately required for the retention of contextual fear [47]. Therefore, in the context of the relation between amygdala rsFC and maternal overprotection, the association with the L amygdala may be linked to diminished responses to positive emotions, whereas the association with the R amygdala may be related to heightened arousal and vigilance in response to negative emotions. Consequently, it appears that the L and R amygdala may cooperate to reinforce the adverse effects of maternal overprotection, potentially contributing to the development and exacerbation of depressive symptoms.

The moderating role of R amygdala–AG rsFC emerged when examining subsequent depressive symptoms, and this effect remained significant after controlling for the time interval between MRI acquisition and CDI‐2 assessment. Maternal overprotection was positively associated with subsequent depressive symptoms across levels of rsFC, with this association being stronger among children with a higher rsFC. Thus, the significant interaction may partly reflect quantitative amplification, whereby higher rsFC intensified the association between perceived maternal overprotection and depressive symptoms. However, the pattern of findings was also suggestive of differential susceptibility, whereby at lower levels of maternal overprotection, children with higher rsFC exhibited lower depressive symptoms relative to those with lower rsFC, whereas this apparent advantage diminished as overprotection increased. In contrast, children with lower rsFC tended to show relatively elevated depressive symptoms even under lower levels of maternal overprotection. These findings align with prior evidence showing decreased rsFC between the R amygdala and the R AG in depressed individuals compared to healthy controls [48] and treatment‐related increases in this connectivity following SSRI intervention in adolescents [49]. Functionally, the R amygdala is involved in rapid and automatic detection of emotionally salient, particularly threatening, stimuli [50], while the R AG plays a role in self‐referential processing and involuntary/exogenous attention [51]. Stronger connectivity between these regions may enhance the integration of emotional salience with self‐relevant meaning, making children more sensitive to caregiving environments. In line with previous studies on differential susceptibility to parenting [52–54], our findings suggest that higher rsFC may confer relative benefits in lower‐risk caregiving contexts while increasing vulnerability in higher‐risk contexts. Nevertheless, because the simple slopes remained positive across rsFC levels, this interpretation should be considered alongside a quantitative amplification account. The convergence of predicted depressive symptoms at high levels of overprotection may also reflect a ceiling effect. Thus, this circuit may both amplify risk‐related associations and index heightened sensitivity to the caregiving environment.

Lastly, we found no significant associations between maternal depressive symptoms and perceived maternal overprotection, amygdala rsFCs, or depressive symptoms in children. This finding suggests that the observed brain–behavior associations may not be directly influenced by maternal depression. This result may be partly because the mothers in our community cohort were generally healthy, with a BDI score of less than 28. Additionally, previous studies have found that the relationship between maternal depression and negative parenting behaviors mostly emphasized on irritability and hostility [22]. Moreover, the timing of depression is a very important moderating factor between maternal depression and parenting [23]. Most studies on the impact of maternal depression on child amygdala rsFC were mainly focused on prenatal and postnatal clinical levels of depression [24].

The study has some limitations to consider. First, perceived parenting was assessed through children’s reports (PBI‐C), as parent‐reported measures were unavailable. Prior research has documented discrepancies between child‐ and parent‐reported parenting, with parents rating themselves as significantly less overprotective than their children report [55, 56]. Future studies incorporating multi‐informant assessments would help evaluate the robustness of these findings. Second, there might be sample bias. We initially had 729 children aged 8.5–10 years in the GUSTO cohort with usable CDI scores (*M* = 11.2 and SD = 7.6), but for this study, we only used data from 220 children with PBI scores and fMRI data. However, we found no significant differences in CDI scores between this sample and the remaining 509 children (*t* = −1.041 and *p* = 0.298), suggesting the current sample represented depressive symptoms across the cohort. A further limitation of the present study is the temporal gap between MRI acquisition and depressive symptom assessment. Specifically, amygdala rsFC was measured at approximately age 7.5 years, whereas CDI‐2 depressive symptoms were assessed 1–3 years later and reflected symptoms experienced only during the preceding 2 weeks. This temporal gap is particularly relevant in this age range, during which rapid neurodevelopment occurs. Therefore, our findings should be interpreted as associations between earlier amygdala rsFC and later depressive symptoms, and future longitudinal studies with repeated neuroimaging and symptom assessments are needed to clarify the temporal dynamics underlying these associations. Additionally, demographic, cultural, and socioeconomic factors may also influence parenting and its neural correlates, which should be considered in future research.

In conclusion, this study examined the relation between maternal parenting and amygdala‐based functional connectivity patterns. The results revealed significant associations with two distinct neural pathways: a positive connection with R BLA–AG rsFC and a negative connection with L SFA–anterior INS rsFC. The former pathway appears to play a pivotal role in the association between maternal overprotection and childhood depressive symptoms, acting as a significant moderator. These findings provide valuable insights into the neurobiological foundations of maternal overprotection and its implication for the development of childhood depression.

## Author Contributions

The study was conceptualized by Lizhu Luo, Ai Peng Tan, and Michael J. Meaney. Data preprocessing was conducted by Lizhu Luo, Shi Yu Chan, and Pei Huang, while Lizhu Luo carried out the formal analysis. The manuscript was initially drafted by Lizhu Luo and subsequently reviewed and revised by Yap Seng Chong, Peter Gluckman, Johan Gunnar Eriksson, Juan Helen Zhou, Ai Peng Tan, and Michael J. Meaney. Project administration and primary funding acquisition were managed by Marielle V. Fortier and Michael J. Meaney.

## Funding

The study is supported by the National Research Foundation (NRF) under the Open Fund‐Large Collaborative Grant (OF‐LCG; Grant MOH‐000504) administered by the Singapore Ministry of Health’s National Medical Research Council (NMRC) and the Agency for Science, Technology and Research (A ^∗^STAR). In RIE2025, the study is supported by funding from the NRF’s Human Health and Potential (HHP) Domain, under the Human Potential Programme. Additional funding was from the Hope for Depression Research Foundation, USA, and the Jacobs Foundation, Switzerland, to Michael J. Meaney. Lizhu Luo received support from the Nanyang Technological University Start‐Up Grant (03INS001883C430); the Ministry of Education, Singapore, under its Academic Research Fund Tier 1 (Grant RS05/24); and the Singapore Ministry of Health through the National Medical Research Council (NMRC) Office, MOH Holdings Pte. Ltd., under the NMRC Population Health Research Grant New Investigator Grant (MOH‐002114). Ai Peng Tan was funded by the NMRC Transition Award (Grant MOH‐001273‐00).

## Conflicts of Interest

The authors declare no conflicts of interest.

## Supporting Information

Additional supporting information can be found online in the Supporting Information section.

## Supporting information


**Supporting Information** Spatial characteristics for the primary cytoarchitectonic amygdala subregions are provided in Table S1. Figure S1: The Brainnetome Atlas (BNA) amygdala subregion masks and their spatial overlap.

## Data Availability

The GUSTO data are not stored in a public repository due to agreements established through multisite partnerships and requirements set by the Internal Review Board. Access to GUSTO data can be granted upon submission and approval of a data access form by the cohort’s executive committee. Upon reasonable request, the corresponding author may provide further details.
