# Supplementary material for: Perceived Maternal Overprotection and Amygdala Functional Connectivity in Children: Implications for Childhood Depressive Symptoms
Source: Depress Anxiety. 2026 Aug 26;2026:4516539. doi: 10.1155/da/4516539 (PMC13507833; doi:10.1155/da/4516539)
Supplement: Supplementary file 1 [file DA-2026-4516539-s001.docx]

**Supplementary Table S1.** Spatial characteristics for the primary cytoarchitectonic amygdala subregions (Voxel Size 1×1×1 mm)

| Regions | Number of Voxels | Volume (mm^3^) |
| --- | --- | --- |
| Centromedial Amygdala, L | 264 | 264 |
| Centromedial Amygdala, R | 329 | 329 |
| Basolateral Amygdala, L | 2441 | 2441 |
| Basolateral Amygdala, R | 2598 | 2598 |
| Superficial Amygdala, L | 1230 | 1230 |
| Superficial Amygdala, R | 1029 | 1029 |

**Supplementary Figure S1.** (A) Masks of amygdala subregions from the Brainnetome Atlas (BNA), including the medial amygdala (MA, blue) and lateral amygdala (LA, green). (B) Spatial correspondence between cytoarchitectonic (blue) and connectivity-based BNA (red) parcellation schemes. The anatomical basolateral amygdala (BLA) shows partial overlap with the BNA lateral amygdala (LA), while the left superficial amygdala (SFA) partially overlaps with the BNA medial amygdala (MA).
